# Emerging Insights into the Distinct Pharmacological Mechanisms of Buprenorphine

**DOI:** 10.1021/acs.jcim.6c00672

**Published:** 2026-05-20

**Authors:** Aràntzazu Alonso-Carrasco, Aleix Quintana-Garcia, Marc Ciruela-Jardí, Verònica Casadó-Anguera, Garett A. Enten, Natàlia Llopart, Berta Carrasco-Martinez, Ning-Sheng Cai, Estefanía Moreno, Sergi Ferré, Vicent Casadó, Leonardo Pardo

**Affiliations:** † Laboratory of Molecular Neuropharmacology, Department of Biochemistry and Molecular Biomedicine, Faculty of Biology and Institute of Biomedicine, University of Barcelona, Barcelona 08007, Spain; ‡ Laboratory of Computational Medicine, Biostatistics Unit, Faculty of Medicine, Autonomous University of Barcelona, Bellaterra 08193, Spain; § Integrative Neurobiology Section, National Institute on Drug Abuse, Intramural Research Program, National Institutes of Health, Baltimore, Maryland 20892, United States

## Abstract

Morphine, an agonist of the μ-opioid receptor (μOR), extracted from *Papaver somniferum* (opium poppy), is an effective analgesic for moderate to severe pain. However, prolonged or inappropriate use of morphine can lead to the development of Opioid Use Disorder (OUD). In contrast, buprenorphine is a high-affinity partial μOR agonist and one of the safest opioids, widely used to treat OUD. This study investigates how specific functional groups of buprenorphine contribute to its strong receptor binding and partial agonist activity. Using alchemical free-energy methods and molecular dynamics simulations, we propose that the 2-hydroxy-3,3-dimethylbutan-2-yl moiety at the C7 position of buprenorphine contributes most to its enhanced binding affinity, while the *N*-cyclopropylmethyl group underlies its partial agonist activity. Moreover, radioligand competitive binding assays revealed that morphine and buprenorphine have nonclassical μOR binding profiles: morphine displays negative cooperativity (binding reduces affinity at the second site), while buprenorphine shows positive cooperativity (binding increases affinity at the second site). These two binding sites are attributed to the two protomers of the μOR homodimer. Molecular dynamics simulations suggest that the *tert*-butyl group of buprenorphine promotes an inward shift of TM 5 by interacting with L221^45.52^ in ECL 2, while the ether bridge causes steric repulsion with I298^6.51^ and V302^6.55^, driving an outward displacement of TM 6. Consequently, the initial binding of buprenorphine to the first protomer induces a conformational rearrangement that is transmitted to the second protomer via the TM5/6 homodimerization interface. These movements act as a positive allosteric mechanism, reshaping the binding pocket of the second protomer to favor an optimal fit of buprenorphine (positive cooperativity). Finding the key molecular characteristics of buprenorphine that drive its beneficial effects is essential to guiding the design of future drugs.

## Graphical abstract

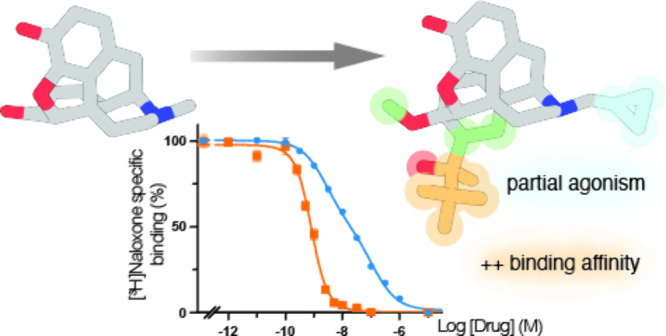

## 1 Introduction

Agonism of the μ-opioid receptor (μOR) offers the most effective treatment for severe pain.^1^ The World Health Organization (WHO) guidelines recommend morphine, an opioid extracted from *Papaver somniferum* (opium poppy), as the analgesic of choice for moderate to severe pain.^2^ For instance, morphine is considered a first-line treatment in the management of cancer pain,^3^ and in cesarean sections, where intrathecal morphine is combined with basic analgesics such as paracetamol and nonsteroidal anti-inflammatory drugs.^4^ Notably, morphine is the only opioid approved by the U.S. Food and Drug Administration (FDA) for intrathecal administration.^5^ However, the Procedure-Specific Postoperative Pain Management (PROSPECT) group highlighted the risks and side effects associated with the use of intrathecal morphine, suggesting that adequate analgesia can be achieved with basic analgesics.^6^

In this context, buprenorphine, a synthetic opioid derived from the alkaloid thebaine,^7^ has emerged as a promising alternative to morphine. Buprenorphine is a partial agonist of μOR, with higher binding affinity than morphine and slow dissociation rate.^8^ Buprenorphine produces analgesia comparable to that of full μOR agonists, displays a ceiling effect on respiratory depression,^9^ and functions as an effective, rapid-acting antidepressant.^10^ It is therefore considered one of the safest opioids.^8,11^ Moreover, its slow dissociation from μOR may contribute to its prolonged therapeutic effect, making it highly effective in the treatment of Opioid Use Disorder (OUD) and a first-line treatment option.^12,13^ Buprenorphine was approved by the FDA for medical use as an analgesic in 1981 and for the treatment of OUD in 2002; however, it is still not authorized for pain management in all European countries.^14^

The diverse alkaloids of *P. somniferum* are categorized into nine classes depending on their chemical scaffold: benzylisoquinoline, morphinan, aporphine, benzo­[c]­phenantridine, papaverrubine, narceine-type, protoberberine, protopine, and phthalideisoquinoline.^15^ Both morphine and buprenorphine belong to the morphinan class and share a similar chemical structure (Figure 1). Compared to morphine, buprenorphine replaces the *N*-methyl group with a longer and bulkier *N*-cyclopropylmethyl group; adds a 2-hydroxy-3,3-dimethylbutan-2-yl moiety (a central quaternary carbon bearing a hydroxyl group and a methylene linker, flanked by two methyl groups, resulting in a highly branched aliphatic side chain) at the C7 position; replaces the hydroxyl group at the C6 position with a methoxy group; and incorporates a specific ether bridge (6,14-endoethano) connecting the C6 and C14 positions, thereby forming a fused tricyclic ring system (Figure 1). This study aims to elucidate the structural determinants within the distinct functional groups of buprenorphine that underlie its enhanced binding affinity and partial agonistic activity.

**Figure 1:**
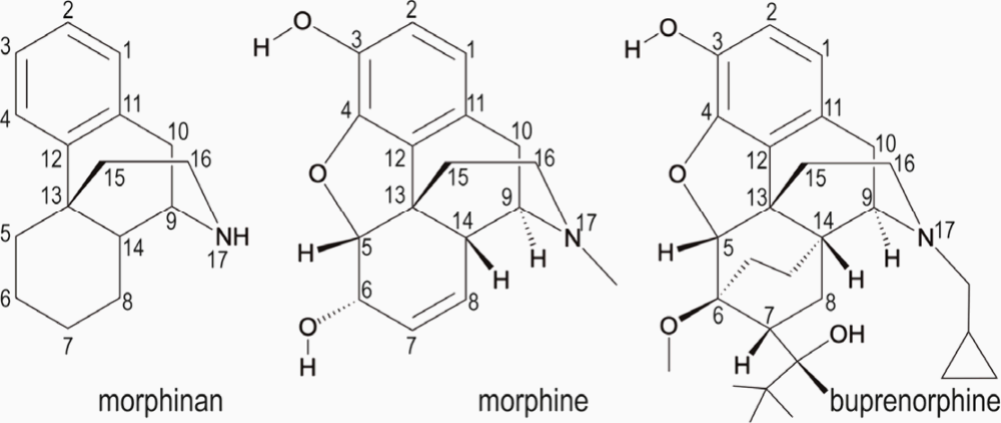
Structures of morphinan, morphine, and buprenorphine.

The μOR belongs to the G protein-coupled receptor (GPCR) family, for which there have been significant advances in structural biology,^16,17^ including the recent elucidation of intermediate conformations along the activation pathway^18^ and conformational preferences associated with engagement of specific downstream signaling transducers.^19^ Among these, and relevant to our study, we would like to highlight the X-ray structures of the inactive μOR bound to a morphinan antagonist^20^ and the active μOR bound to a morphinan agonist,^21^ as well as the cryo-EM structure of active μOR-Gi bound to morphine.^22^ Comparison of inactive and active μOR revealed a common rearrangement in the packing of three conserved amino acids (the PIF motif) that transmits the signal from the orthosteric binding site to the G protein binding site.^21,23,24^ Using this novel structural knowledge, we employed alchemical free-energy methods and molecular dynamics (MD) simulations to evaluate how distinct functional groups of buprenorphine contribute to its enhanced binding affinity and partial agonist activity, as measured by radioligand competitive binding, cAMP, and NanoLuc Binary Technology (NanoBiT) assays.

## 2 Materials and Methods

### 2.1 Cell Culture and Stable Cell Lines

Human embryonic kidney 293 T (HEK-293 T) cells were obtained from ATCC (American Type Culture Collection, Rockville, MD, USA; CRL-321, RRID: CVCL_0063) and grown in Dulbecco’s modified Eagle medium (DMEM) supplemented with 10% (v/v) heat-inactivated fetal bovine serum (Sigma-Aldrich, Sant Louis, MO, USA), 100 U/ml penicillin, 100 μg/mL streptomycin, MEM nonessential amino acid solution (1/100) and 2 mM l-glutamine (all supplements from Gibco, Thermo Fisher Scientific). Cells were cultured in CytoOne (USA Scientific, New York, NY) treated culture plates in a Forma Series II Water Jacket incubator (Thermo Fisher Scientific, Waltham, MA) at 37 °C, 5% CO2, 90% humidity.

In addition to routine cell culture conditions, stable cell lines were generated as described below. The cDNA for the human μOR was obtained from the Missouri S&T cDNA Resource Center and was N-terminally modified with an in-frame fusion of a signal peptide to enhance cell-surface expression,^25^ followed by a Flag epitope tag and subcloned into the pcDNA5/FRT plasmid. The construct was confirmed by sequence analysis. This plasmid was cotransfected into Flp-In HEK-293T cells with the Flp recombinase expression vector pOG44 (1 μg/9 μg) to generate FLP-FRT-HEK stable cell lines expressing the μOR. Transfection was performed using the Lipofectamine method, according to the manufacturer’s instructions (Invitrogen, Thermo Fisher Scientific). Clones resistant to hygromycin B (50 μg/mL) were isolated, and a cell line expressing μOR (μOR cells) was selected based on a significant functional response to the μOR agonist endomorphin-1, as measured by a DMR assay.^26^

### 2.2 Radioligand Binding Assays

μOR cell suspensions were disrupted using a Polytron homogenizer (PTA 7 rotor, setting 3; Kinematica, Basel, Switzerland) for three 5 s periods in 10 volumes of 50 mM Tris-HCl buffer (pH 7.4) containing a protease inhibitor cocktail (Sigma-Aldrich, St. Louis, MO, USA). Membranes were obtained by centrifugation at 105000 g for 45 min at 4 °C. The pellet was stored at −80 °C until the test day, washed once more as described above, and resuspended in 50 mM Tris-HCl buffer for immediate use. Membrane protein concentration was quantified by the bicinchoninic acid method (Pierce Chemical Co., Rockford, IL, USA) using bovine serum albumin dilutions as standard. Binding experiments were performed with membrane suspensions (0.2 mg of protein/mL) at room temperature in 50 mM Tris-HCl buffer (pH 7.4) containing 5 mM MgCl_2_.

For competitive binding assays, membrane suspensions were incubated for 2 h with a constant free concentration of 2.4 nM [^3^H]­naloxone (PerkinElmer, Waltham, Massachussets, USA) and increasing concentrations (1 pM to 10 μM) of the μOR agonists morphine (Tocris Bioscience, Bristol, UK) or buprenorphine (British Pharmacopoeia Commission Lab). Nonspecific binding was determined using 30 μM of naloxone. Free and membrane-bound ligands were separated by rapid filtration of 400 μL aliquots using a cell harvester (Brandel, Gaithersburg, MD, USA) through Whatman GF/C filters presoaked in 0.3% polyethylenimine, followed by a 5 s wash with 5 mL of ice-cold 50 mM Tris-HCl buffer. The filters were incubated overnight at room temperature in 5 mL of Ultima Gold MV scintillation cocktail (PerkinElmer), and radioactivity was measured using a Tri-Carb 2900 scintillation counter (PerkinElmer) with an efficiency of 62%.

### 2.3 Radioligand-Binding Data Analysis

Radioligand competition curves were analyzed by nonlinear regression using GraFit curve-fitting software (Erithacus, UK), by fitting the binding data to the mechanistic two-state dimer receptor model, as described in detail elsewhere.^27,28^ Goodness of fit was assessed based on the reduced χ2 value provided by the nonlinear regression program. The test of significance for two different model population variances was based on the F distribution (see Casadó-Anguera et al.^29^ for details). According to the F test (*p* < 0.05), the more complex model (positive or negative cooperativity) fits better that the simpler one state model (noncooperativity). This yields two macroscopic dissociation constants that describe the affinity of the competing ligand for protomers 1 (K_DB1_) and 2 (K_DB2_). Results are reported as parameter values ± SEM of different independent experiments.

### 2.4 Constructs

For NanoBiT assays, human μOR and Gαo cDNA were cloned in the pIREShyg3 plasmid vector within the Afe I and Xba I restriction enzyme sites (Clontech Laboratories, Mountain View, CA) containing the sequences for the small subunit (SmBiT) and long subunit (LgBiT) of nanoluciferase,^30^ respectively (pIRES-HA-PS-MOR^SmBiT^, pIRES-HA-Gαo^LgBiT^). pIRES-HA^LgBiT^ was utilized as an empty vector negative control. MOR included the mGlu5 receptor signal peptide in 5′ of the MCS to allow for plasma membrane trafficking. All constructs were HA tagged for detection and verified by DNA sequencing.

### 2.5 NanoLuc Binary Technology Assay

HEK293T cells were plated in 6-well plates and transiently transfected with 1.5 μg pIRES-HA-PS-μOR^SmBiT^ and 1.5 μg pIRES-HA-Gαo^LgBiT^ together using polyethylenimine (PEI) transfection reagent.^31^ Cells transfected with 1.5 μg pIRES-HA-PS-hμOR^SmBiT^ and 1.5 μg pIRES-HA^LgBiT^ were utilized as a negative control. Media in each well was replaced with fresh DMEM after 4 h and cells were left in the incubator overnight. The next morning, cells were replated to 96-well poly-d-lysine precoated plates and allowed to incubate overnight. The following day, media was replaced with 0.1% glucose/2 mM NaHSO_3_/PBS containing 100 pM to 1 μM of morphine or buprenorphine and incubated for 15 min at 37 °C. Coelenterazine H (MedLumine, Northfeild, IL) was subsequently added in a final concentration of 5 μM and plates were incubated at 37 °C for 5 min. Luminescence was then measured at 485 nm in a Mithras LB 940 plate reader (Berthold Technologies, Wildbad, Germany). Net luminescence was calculated by subtracting mean luminescence of wells containing untreated cells transfected with pIRES-HA-PS-hμOR^SmBiT^ and the empty vector control (pIRES-HA^LgBiT^) from the luminescence recorded for each treated well. Results are expressed as percentage of the maximal net luminescence of cells treated with morphine (RLU % Emax morphine).

### 2.6 cAMP Accumulation Assay

cAMP was determined using the Lance Ultra cAMP kit (PerkinElmer), which is based on homogeneous time-resolved fluorescence energy transfer technology. Briefly, μOR cells (1200–1500 per well), growing in medium containing 50 μM zardaverine (Tocris #1046) as phosphodiesterase inhibitor, were incubated for 15 min in white ProxiPlate 384-well microplates (PerkinElmer) at 25 °C with vehicle or morphine or buprenorphine (doses ranging from 0.0001 to 10 μM) before adding forskolin (0.5 μM, Tocris, Bristol, UK, #1099) and incubating for 15 additional min. Every condition was assayed in quadruplicates within each individual experiment. Fluorescence at 665 nm was analyzed on a PHERAstar Flagship microplate reader equipped with an HTRF optical module (BMG Lab Technologies, Offenburg, Germany).

### 2.7 Initial μOR Models

The active μOR-morphine-G_i_ cryo-EM structure (PDB id 8EF6)^22^ and the inactive μOR-μOR homodimer with the TM5/6 interface (4DKL)^20^ were used. Protonation states were assigned with the PDB 2PQR tool^32^ using PROPKA to predict the p*K*_a_ values of ionizable groups in the proteins at pH 6.5.^33^ Disulfide bonds between cysteines were built using the tleap module of Ambertools19. Buprenorphine was docked into the orthosteric binding cavity using the morphine structure as a template. In the μOR−μOR homodimer, one of the protomers binds morphine or buprenorphine, while the other remains unliganded. These systems were oriented by the Orientations of Proteins in Membranes (OPM) database^34^ and embedded in a lipid bilayer box, constructed using PACKMOL-memgen,^35^ containing 1-palmitoyl-2-oleoyl-*sn*-glycero-3-phosphocholine (POPC), water molecules (TIP3P), and monatomic Na^+^ and Cl^–^ ions (0.15 M). It is important to note that GPCR conformations can be highly sensitive to membrane composition;^36^ however, only a POPC bilayer was considered in this study. The resulting systems comprise 250k atoms in a box of ∼ 125 Å × 126 Å × 156 Å for the μOR-G_i_ complex and 140k atoms in a box of ∼127 Å × 127 Å × 114 Å for the μOR-μOR homodimer (see the Supporting Information in the Zenodo repository for detailed values).

### 2.8 Molecular Dynamics Simulations

MD simulations of the monomeric μOR-G_i_ system were performed with GROMACS 2024.2 ^37^ and with AMBER24 ^38^ for the dimeric μOR-μOR system using the protocols previously reported.^39,40^ The amber14sb-ildn force field was used for the protein, solvent and ions,^41^ a GROMACS adaptation of lipid14 for lipids,^42^ and the general Amber force field (GAFF2) with HF/6–31G*-derived RESP atomic charges for morphine and buprenorphine.^43^ MD trajectories were produced at a temperature of 300 K with separate a v-rescale thermostat for the receptor, ligand, lipids and solvent molecules in GROMACS or the Langevin dynamics thermostat in AMBER. The Berendsen barostat was chosen for equilibration and Parrinello–Rahman for production MDs in GROMACS and the Monte Carlo barostat in AMBER. A time step of 2.0 fs was used for the integration of equations of motions. All bonds and angles involving hydrogen atoms were constrained using the LINCS algorithm in GROMACS and SHAKE in AMBER. All other bonds and angles were left unconstrained, and hydrogen mass repartitioning was not employed. Lennard-Jones interactions were computed using a cutoff of 1.1 nm under the Verlet cutoff scheme for neighbor searching, and the electrostatic interactions were treated using PME with the same real-space cutoff under periodic boundary conditions. Center of mass motion was removed from all system. Molecular systems were subjected to 5000 steps of energy minimization, using the steepest descent algorithm, PME electrostatics with Verlet cutoff-scheme. This was followed by a 25 ns equilibration protocol consisting of 6-steps, in which positional restraints are progressively removed, from all heavy atoms to only helix Cα carbons being restricted, meanwhile gradually reducing the applied forces, from 1000 kJ mol^–1^ nm^–2^ to 0 kJ mol^–1^ nm^–2^. After equilibration, three replicas of 1 μs unrestrained MD trajectory were generated for the monomeric μOR-G_i_ system and three replicas of 4 μs for the dimeric μOR-μOR system. For complete details, see Supporting Information in the Zenodo repository.

### 2.9 Nonequilibrium Thermodynamic Integration

A series of chemically similar intermediate structures connecting buprenorphine to morphine were generated and parametrized as described in Section 2.8. To perform nonequilibrium transitions employing a single topology, hybrid structures and topology for each consecutive ligand pair were generated using pmx and its mapping tool.^44,45^ Each ligand pair was simulated as a single hybrid structure in physical states A and B, both in solution and in complex with active μOR. Systems were energy-minimized, equilibrated for 5 ns in the NPT ensemble at 298 K in a 5-step protocol, and then simulated for 6 ns in the NPT ensemble at 298 K and 1 bar. After discarding the first 2 ns for relaxation, 80 evenly spaced snapshots were extracted from each trajectory. From these configurations, alchemical transitions between states A and B (and vice versa) were performed over 50 ps each, and the work associated with each transition was obtained from the derivative of the Hamiltonian with respect to the λ coupling parameter. The free energy estimate (Δ*G* ^TI^) of a given transition and its uncertainty were calculated from the overlap of forward and backward work distributions using the Crooks Fluctuation Theorem,^46^ as implemented in pmx. For transitions in which poor overlap was obtained, the transition time was increased to 500 ps (see Supplementary Figure S1). Finally, the relative ΔΔ*G* ^TI^ was calculated by subtracting the mean of Δ*G* ^TI^ values in solution from those in complex with μOR. The reported error for each ΔΔ*G* ^TI^ corresponds to the square sum of standard deviation of the mean for three replicates and the error of each Δ*G* ^TI^. This procedure corresponds to 30 ns of MD simulation per Δ*G* ^TI^, totaling 180 ns per ΔΔ*G* estimate for a three-replica run.^47^

### 2.10 MD Analysis and Data Visualization

The analysis of the trajectories was performed using MDAnalysis,^48^ cpptraj,^49^ and GetContacts (https://getcontacts.github.io/); and visualization and image rendering were performed with PyMOL^50^ and VMD.^51^

### 2.11 Statistical Analysis

Comparisons among groups were performed by one-way ANOVA followed by multiple comparison post hoc test (as indicated in figure legends), only if F in ANOVA achieved *p* values of <0.05 and there was no significant variance inhomogeneity. A *p* value of <0.05 was considered significant. GraphPad Prism 10.6 software (San Diego, CA, USA) was used for data analysis.

## 3 Results and Discussion

### 3.1 Radioligand Binding Assays

Competitive inhibition experiments using the radiolabeled μOR antagonist [^3^H]­naloxone with increasing concentrations of morphine and buprenorphine (Figure 2) were conducted on membrane preparations from a previously described and characterized HEK-293T cell lines stably expressing μOR (see Methods). As previously observed for the endogenous μOR agonist endomorphin-1 in competitive inhibition experiments of [^3^H]­naloxone using the same cell line,^26^ morphine also displayed biphasic curves that provided a significantly better fit than monophasic curves (*p* < 0.05, using the F-test; see Methods), indicating the existence of two binding sites with different affinities on the same target. In most cases, the presence of two affinity sites arises from negative cooperativity in agonist binding at the two orthosteric sites of a GPCR homodimer, where binding of the ligand to the first protomer decreases the affinity of the binding to the second protomer.^52^ In fact, μOR homodimerization has been demonstrated with diverse biophysical techniques, more recently including single-fluorescent molecule tracking analysis^53^ (see Discussion). Accordingly, morphine bound to the μOR with two distinct affinities, exhibiting negative cooperativity (*K*_DB1_ = 2.2 ± 0.7 nM; *K*_DB2_ = 70 ± 20 nM). In contrast, buprenorphine did not follow a classical monophasic curve but instead showed an abrupt shift consistent with positive cooperativity (*K*_DB1_ = 0.9 ± 0.1 nM; *K*_DB2_ = 0.3 ± 0.1 nM), which likewise required a dimer-receptor model for an optimal fit. Therefore, occupancy of the first protomer by morphine reduces its affinity for the second protomer (negative cooperativity, dimer cooperativity index D_C_ of −0.9), whereas occupancy of the first protomer by buprenorphine enhances the affinity of the second protomer for the ligand (positive cooperativity, D_C_ of +1.0). Moreover, the drug concentration that occupies 50% of the μOR protomers (B_50_)^27^ is 0.5 nM for buprenorphine and 12 nM for morphine. Since each ligand has two affinity constants, B_50_ provides a more global view, showing that buprenorphine half-occupies μORs at a concentration 24 times lower than morphine.

**Figure 2:**
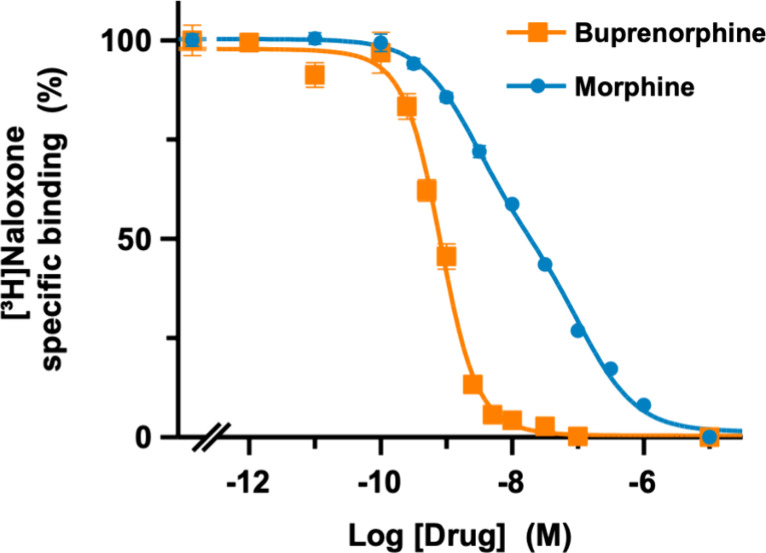
Competitive inhibition curves of [^3^H]­naloxone with increasing concentrations of morphine or buprenorphine in HEK-293 T cells stably expressing μOR. Data was adjusted with the mechanistic dimer-receptor model (*K*_DB1_ and *K*_DB2_; *n* = 4, with triplicates), which provided a significantly better fit compared with the simpler one-state model for both ligands (*p* < 0.05 in all cases; see Materials and Methods).

### 3.2 Mechanism Underlying the Positive Cooperativity of Buprenorphine

To characterize the different molecular signatures of morphine and buprenorphine at the μOR, we performed three independent 1 μs unbiased MD simulations of each ligand bound to the μOR-Gi complex (see Methods). Figure 3a,b shows the evolution of morphine and buprenorphine within the orthosteric binding site of the μOR in a representative simulation. The stabilities of these ligands in the simulations were analyzed via root mean-square deviations (rmsd) of the heavy atoms (Figure 3a,b). Predicted contacts, as calculated with the GetContacts software, of morphine and buprenorphine with the μOR (Figure 3c), along with detailed views of these interactions (Figure 3a,b) are shown. Notably, buprenorphine forms additional contacts, relative to morphine, with I146^3.29^, L221^45.52^, K235^5.39^, V238^5.42^, W295^6.48^, I298^6.51^, H299^6.52^, V302^6.55^, W320^7.35^, I324^7.39^, G327^7.42^, and Y328^7.43^ (Figure 3d).

**Figure 3:**
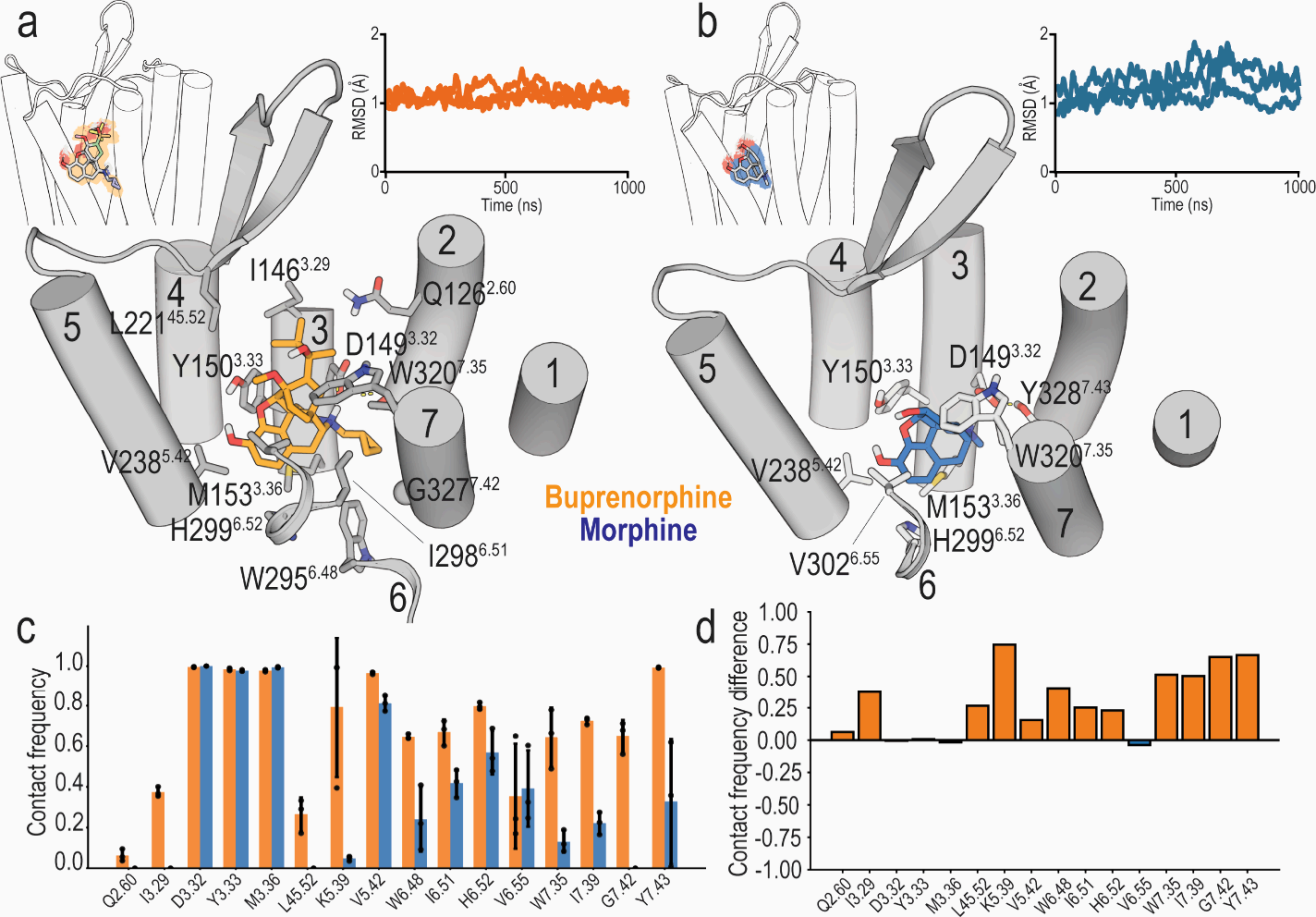
MD simulations of the μOR-Gi complex bound to morphine and buprenorphine. (a, b) Representative structures (solid sticks) and evolution (lines) of buprenorphine (a, in orange) and morphine (b, in blue) in complex with the μOR (white cylinders, only the initial structure is shown), as devised from MD simulations. The stability of the ligand–receptor complexes was analyzed via rmsd of the heavy atoms of buprenorphine (a) and morphine (b) during three replicas of 1 μs unbiased MD simulations. Detailed views of buprenorphine (a) and morphine (b) and their receptor interacting side chains. (c) Calculated frequency (%) of noncovalent contacts (hydrogen bond, hydrophobic, and van der Waals interactions, as described in https://getcontacts.github.io/interactions.html) between side-chain residues of μOR involved in stable interactions with buprenorphine or morphine during MD simulations. (d) The contact frequency differences between buprenorphine and morphine are also shown.

The simulations show that buprenorphine alters the conformation of TMs 5 and 6 with respect to morphine (Figure 4a). The bulky and rigid ether bridge linking the C6 and C14 positions of the buprenorphine morphinan scaffold induces an outward displacement of TM 6 in the μOR due to steric clashes (see below) with the β-branched, and thus also bulky and rigid, residues I298^6.51^ and V302^6.55^ (Figure 4b). This clash is similar to that observed between the bulky tricyclic moiety of clozapine and TM 5 of D_2_R.^54^ On the other hand, the also bulky *t*-butyl group of the 2-hydroxy-3,3-dimethylbutan-2-yl moiety at the C7 position of buprenorphine induces a movement of ECL 2 to interact with L221^45.52^ and I146^3.29^, triggering an inward displacement of TM 5 (Figure 4c).

**Figure 4:**
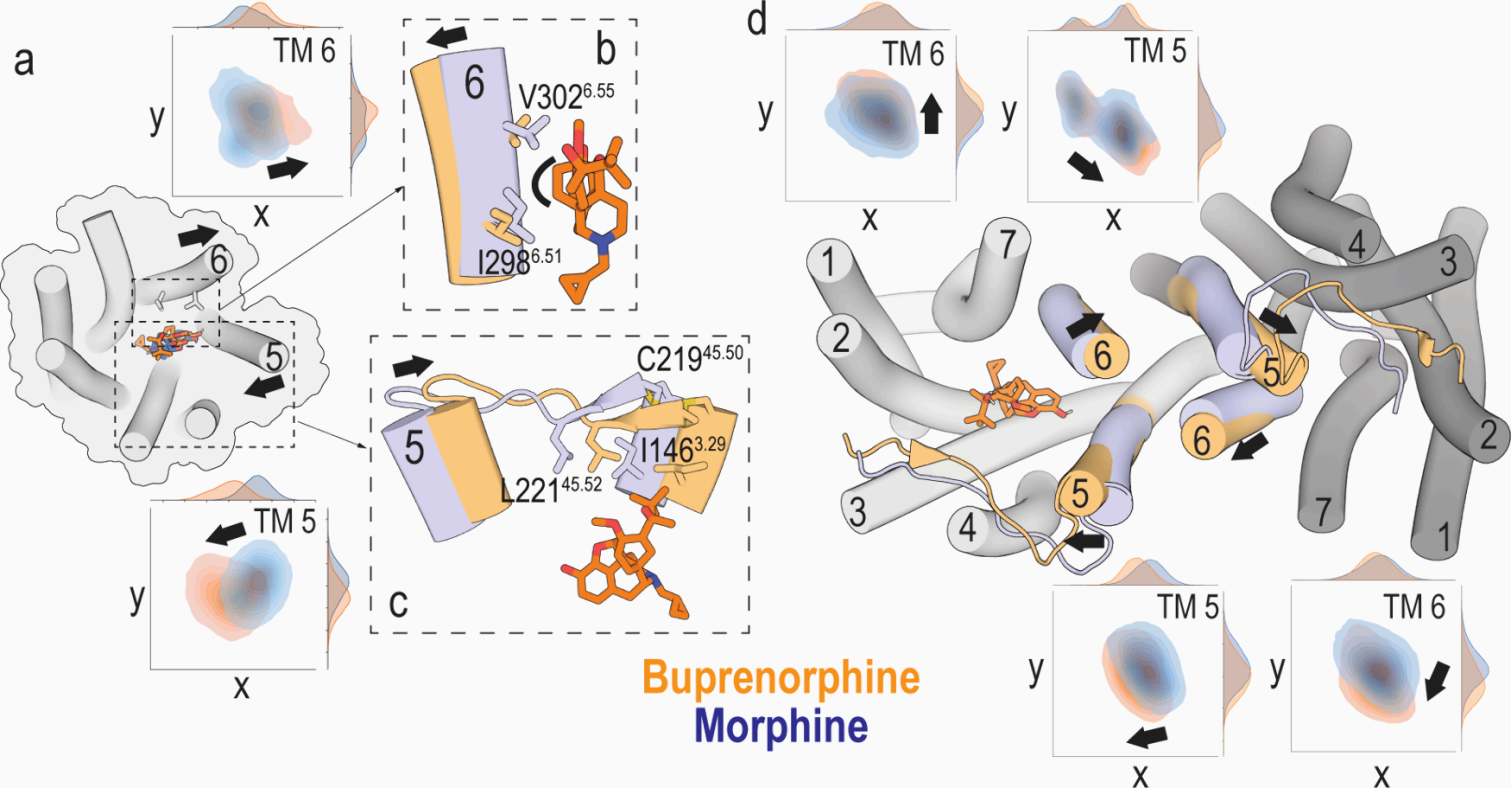
Influence of buprenorphine binding to μOR in the conformation of TMs 5 and 6. (a) Contour plots of the evolution of the center of mass of amino acids E231^5.35^-K235^5.39^ in TM5 and Y301^6.54^-L307^6.60^ in TM6 of μOR bound to morphine (blue) and buprenorphine (orange) during three replicas of unbiased 1-μs MD simulations. The *xy* plane is defined by the Orientations of Proteins in Membranes (OPM).^34^ Distributions of the *x* and *y* values are shown on the top *x*-axis and right *y*-axis, respectively. (b) Detailed view of the outward movement of TM 6 due to the steric clash (solid black curve) between the C6–C14 ether bridge of buprenorphine and I298^6.51^ and V302^6.55^. (c) Detailed view of the inward movement of TM 5 caused by the interactions of the C7 *t*-butyl group of buprenorphine with L221^45.52^ in ECL 2 and I146^3.29^ in TM 3. The disulfide bridge between TM 3 and ECL 2 is shown. Note that the orientation of the molecular models in panels B and C do not correspond to that shown in panel A. (d) Representative structures obtained in three replicas of unbiased 4-μs MD simulations of the μOR-μOR homodimer. Contour plots depicting the evolution of TMs 5 and 6, calculated as in panel a, for the protomer bound to morphine or buprenorphine (left) and the unliganded protomer (right). The movement of TMs 5 and 6 forming the TM5/6 interface and ECL 2 is shown, whereas the other TM helices correspond to the initial model (in light and dark gray for protomers A and B, respectively). Black arrows represent the movement of TM helices in the presence of buprenorphine relative to morphine.

The molecular interface through which the μOR protomers interact is well characterized. First, the crystal structure of the inactive μOR revealed a mode of receptor homodimerization, occurring precisely via TMs 5 and 6.^20^ In this assembly, TMs 5 and 6 of protomer A form a four-helix bundle with TMs 5 and 6 of protomer B. Second, bimolecular fluorescence complementation (BiFC) and total internal reflection fluorescence (TIRF) microscopy experiments, in combination with disruptive peptides targeting a specific dimer interface (reviewed in Franco et al.^55^), revealed that the μOR homodimer interface is formed by TMs 5 and 6 (TM5/6 interface).^28^ Thus, the movements of TMs 5 and 6 in protomers A and B are not independent, but rather tightly coupled. To investigate these concerted movements, we examined the conformation of the TM5/6 interface of the inactive μOR−μOR homodimer (one protomer binds morphine or buprenorphine, while the other remains unliganded) across three replicas of unbiased 4-μs MD simulations (see Methods). Longer simulation times than those used for the μOR-Gi complex were employed due to the high stability of the four-helix bundle forming the TM5/6 interface, which restricts ligand-induced conformational changes. Clearly, buprenorphine binding to protomer A induces an inward displacement of TM 5 and an outward displacement of TM 6 in protomer B, while in turn drive the opposite movements in protomer B, outward TM 6 and inward TM 5, respectively (Figure 4d). Thus, the trends observed in the μOR-Gi simulations (Figure 4a) are reproduced in the μOR-μOR simulations (Figure 4d). Buprenorphine binding to the first protomer requires energy to move these helices, whereas in the second protomer, these movements have already occurred, explaining the positive cooperativity. We hypothesize that buprenorphine bound to protomer A of the μOR acts as a positive allosteric modulator, enhancing the binding affinity of buprenorphine to protomer B through allosteric modification of the receptor’s binding cavity.

### 3.3 Alchemical Free Energy Calculations of Ligand Binding

Binding affinity calculations using MD simulations and alchemical (nonphysical) thermodynamic cycles are valuable for assessing the contribution of specific ligand groups to binding affinity.^47,56,57^ This thermodynamic cycle enables the indirect calculation of free energy differences between two ligands by using computationally tractable transformation paths (Figure 5a). In the alchemical transformation, one molecule (buprenorphine) is gradually converted into another (morphine). Because the structural differences between buprenorphine and morphine are substantial for this type of transformation, four intermediate states, designated A1, A2, A3, and A4, were introduced (Figure 5b), to improve convergence and accuracy.^58^ Atom mapping was established according to predefined rules to minimize perturbations and maintain system stability during simulations. Relative binding free energy differences are calculated as detailed in section 2.9 and their convergence is assessed based on the overlap between Gaussian-fitted work distributions (Supplementary Figure S2). In A1, the N-cyclopropylmethyl group of buprenorphine was replaced by a *N*-methyl. Deletion of the cyclopropyl moiety results in a theoretical binding free energy difference of ΔΔ*G* ^TI^_Bupre→A1_ = 1.34 ± 0.99 kcal/mol (Figure 5b). The cyclopropyl moiety of buprenorphine stabilizes the interaction with μOR by inserting into a polar cavity that extends toward the intracellular side,^21^ which is delimited by the small side chain of G327^7.42^ (Figure 5c). In this cavity, the cyclopropyl moiety primarily interacts with the highly polarizable sulfur atom of M153^3.36^, which can form stronger interactions than aromatic or hydrophobic side chains,^59^ and with the aromatic side chains of W295^6.48^ and Y328^7.43^ (Figure 5c). In the A1 → A2 transition, the *t*-butyl group in the 2-hydroxy-3,3-dimethylbutan-2-yl moiety at the C7 position was replaced by a hydrogen atom. The free energy penalty for this deletion is ΔΔ*G* ^TI^_A1→A2_ = 1.63 ± 1.11 kcal/mol, making it the largest among all transitions (Figure 5b). This group moves ECL 2 (see above) to facilitate hydrophobic interactions with I146^3.29^ and L221^45.52^ (Figure 5c). In A3, the remaining part of the 2-hydroxy-3,3-dimethylbutan-2-yl moiety of buprenorphine, specifically the central quaternary carbon bearing a hydroxyl group and a methyl group in A2, was replaced by a hydrogen atom. The free energy penalty of this A2 → A3 transition is ΔΔ*G* ^TI^_A2→A3_ = 1.14 ± 0.58 kcal/mol, resulting from the loss of the hydrogen bond interaction between the hydroxyl group and Trp320^7.35^ (Figure 5c). Significantly, the 2-hydroxy-3,3-dimethylbutan-2-yl moiety at the C7 position of buprenorphine contributes most to the enhanced binding affinity. The free energy associated with the removal of this group (A1 → A2 + A2 → A3 transitions) can be estimated as ΔΔ*G* ^TI^_A1→A3_ = 1.63 + 1.14 = 2.77 ± 1.25 kcal/mol. In the A3 → A4 transition, the methoxy group at the C6 position was replaced by a hydroxyl group, with ΔΔ*G* ^TI^_A3→A4_ = 0.45 ± 0.34 kcal/mol (Figure 5b), the smallest value among all transitions. The methoxy group is predicted to contact the amphipathic K235^5.39^ side chain and V302^6.55^. Finally, the ether bridge connecting the C6 and C14 positions was removed in the A4 → Morph transition. ΔΔ*G* ^TI^_A4→Morph_ is −1.52 ± 0.38 kcal/mol, representing the only perturbation that favors morphine over buprenorphine. This negative value led us to define the interaction with I298^6.51^ and V302^6.55^ as a steric clash (see above). Summing all these transition yields a final theoretical ΔΔ*G* ^TI^_Bupre→Morph_ value of 3.03 ± 1.67 kcal/mol. Comparison of this theoretical free energy with experimental values obtained from radioligand binding assays is not straightforward, owing to the presence of two experimentally determined dissociation constants, *K*_D1_ and *K*_D2_. Using *K*_D1_, the resulting ΔΔ*G* is 0.6 kcal/mol, whereas using *K*_D2_, it is 3.4 kcal/mol. The appropriate method for averaging these values remains unclear; however, the theoretical value falls within the range of experimentally obtained results. Importantly, this theoretical free energy is in good agreement with earlier measurements showing that buprenorphine has ∼200-fold higher affinity (∼3.3 kcal/mol) for the μOR than morphine, as determined by displacement from rat spinal cord receptors.^60^

**Figure 5:**
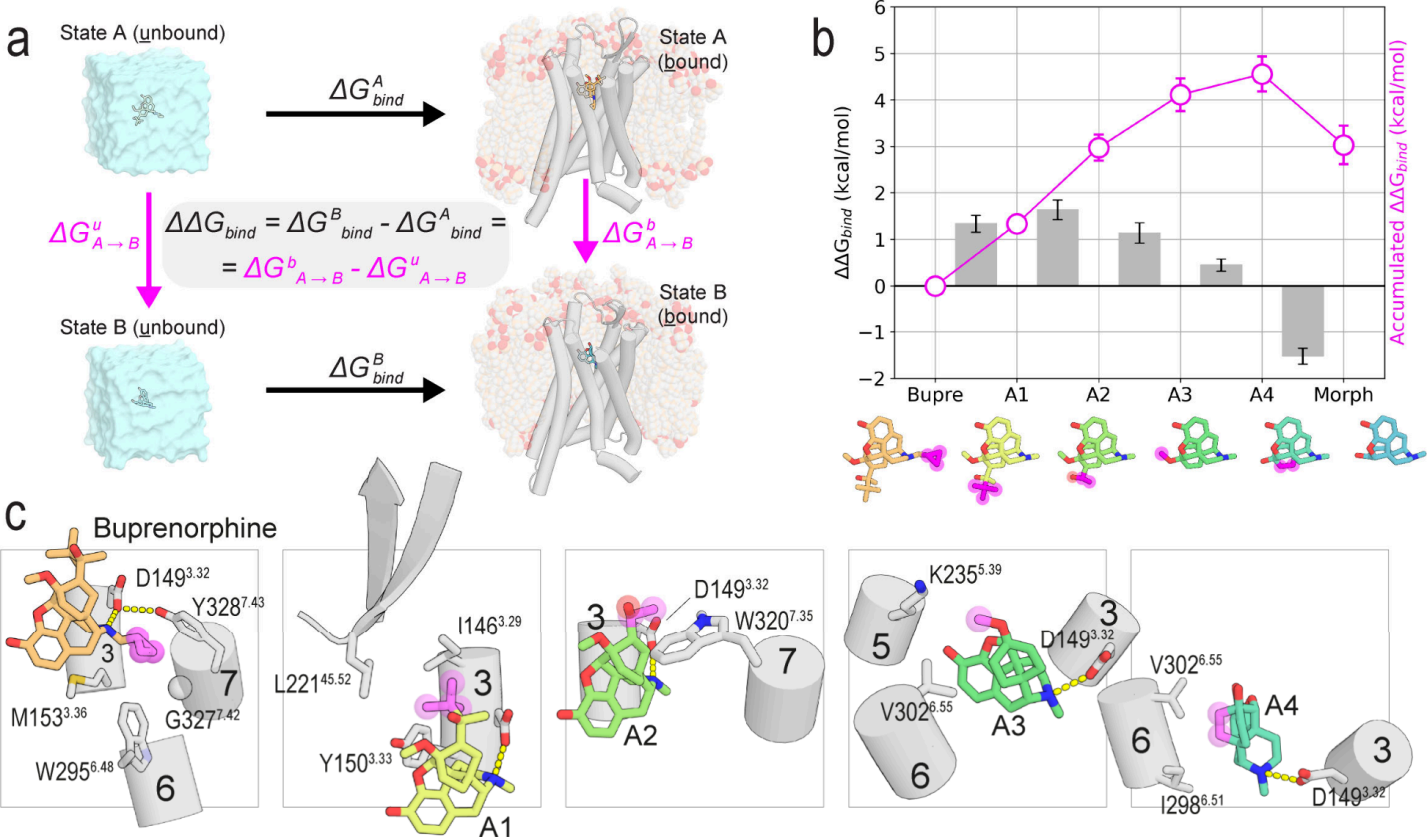
Alchemical free energy calculations. (a) Thermodynamic cycle on which nonequilibrium TI relative free energy calculation is based. Horizontal black arrows represent physical binding processes of ligands A and B (Δ*G* ^A^_bind_ and Δ*G* ^B^_bind_), while vertical violet arrows depict alchemical transformations in the bound and unbound states (Δ*G* ^b^_A→B_ and Δ*G* ^u^_A→B_). The free energy difference between two ligands can be calculated as ΔΔ*G*_bind_ = Δ*G* ^B^_bind_ – Δ*G* ^A^_bind_ (involved and computationally expensive) or ΔΔ*G*_bind_ = Δ*G* ^b^_A→B_ – Δ*G* ^u^_A→B_ (simple and computationally accessible). (b) Calculated ΔΔ*G*_bind_ values for each ligand pair (gray bars), along with the accumulated ΔΔ*G*_bind_ relative to buprenorphine (violet line). Shown below are the 3D structure of buprenorphine (Bupre), morphine (Morph), and intermediates A1 to A4. Structural differences undergoing alchemical transmutation are highlighted in violet spheres. (c) Detailed view of the interactions between the transmuted functional groups (violet spheres) and μOR, with hydrogen bonds with D149^3.32^ depicted as dashed yellow lines for reference.

### 3.4 Buprenorphine Acts As a Partial Agonist

First, we evaluated agonist-induced real-time functional coupling of μOR to Go using an engineered NanoBiT assay (see Methods), as previously described.^61^ μOR-SmBiT was transiently expressed in HEK-293 T cells together with Gαo-LgBiT (Figure 6a). Subsequently, the morphine- and buprenorphine-induced increases in coelenterazine-induced NanoLuc luciferase bioluminescence were recorded (Figure 6b). As expected from previous studies indicating the partial agonism of buprenorphine,^62^ the resulting sigmoidal dose–response curves confirmed a statistically significant decrease in Emax for buprenorphine compared to morphine (Figure 6c), with no significant differences in pEC_50_ (Figure 6d). Second, we evaluated agonist-induced signaling response in cAMP production (Figure 6e). Cells stimulated with forskolin and treated with morphine or buprenorphine showed reduced cAMP production, as expected for Gi-coupled receptors (Figure 6f). Morphine behaved as a full μOR agonist, reducing intracellular cAMP levels to 97 ± 4% of maximal inhibition, a response comparable to that produced by endomorphin-1 (100 ± 2%). In contrast, buprenorphine acted as a partial agonist, inducing a significantly smaller inhibition of cAMP accumulation (80 ± 3%, *p* < 0.01), with no significant differences in pEC_50_ (Figure 6h).

**Figure 6:**
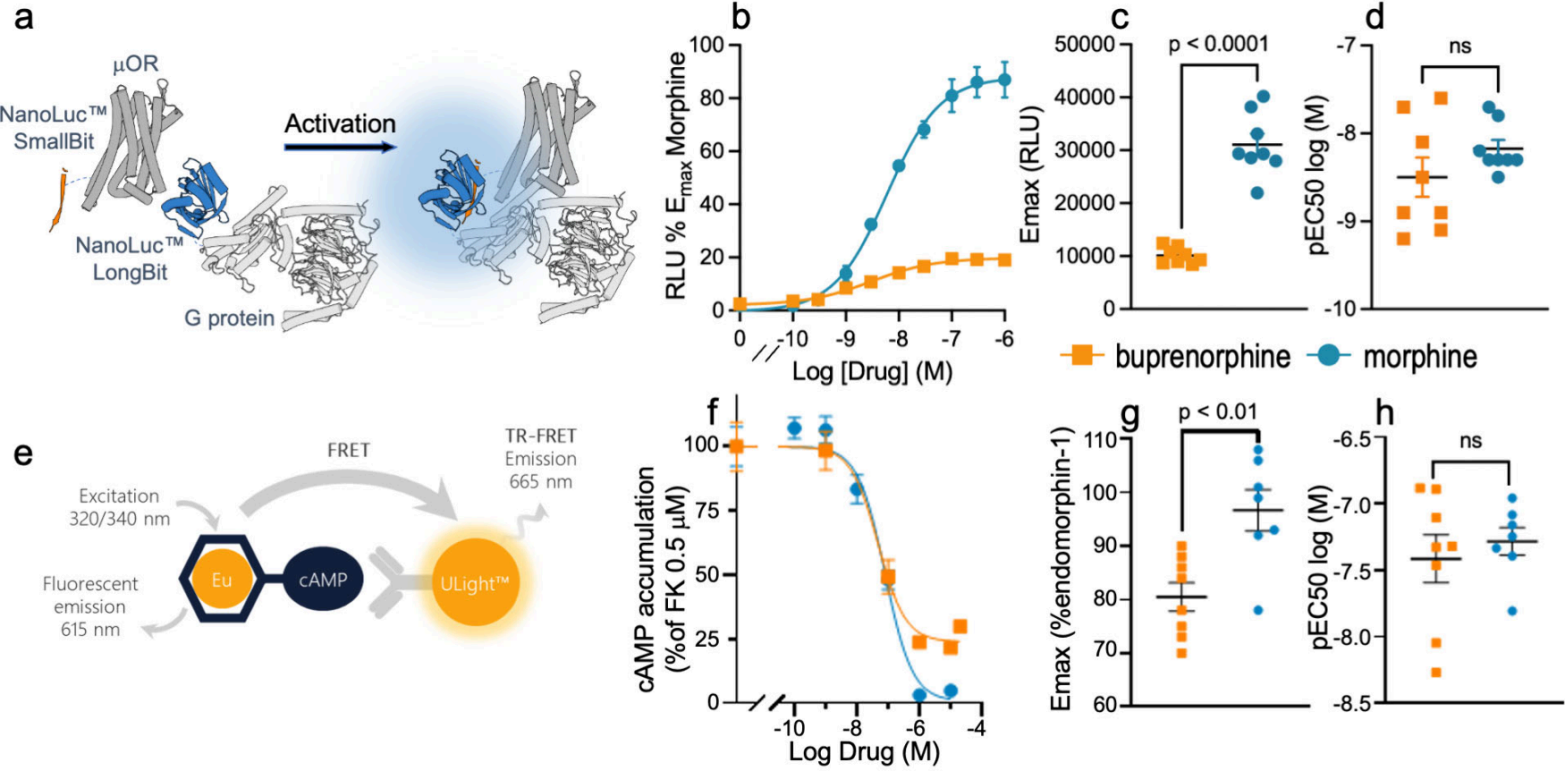
Functional characterization of morphine and buprenorphine at the μOR in transfected HEK-293T cells. (a) Schematic representation of the NanoBiT assay. Nanoluciferase (NL) SmBiT and LgBiT complementary fragments are fused to μOR and Go, respectively. As the agonist induces the coupling of the receptor to Gαo, the NL subunits reconstitute an active NL, which becomes a reporter of receptors’ coupling. (b) Representative concentration–response curves showing morphine- and buprenorphine-induced increases in coelenterazine-driven NanoLuc luciferase bioluminescence. Results were normalized to the maximal Relative Luminescence Units (RLU) produced by morphine (100%). (c, d) *E*_max_ (c) and pEC_50_ (d) of the NanoBiT assay with morphine and buprenorphine (in means ± S.E.M.; *n* = 8 in all experiments, with triplicates). (e) Schematic representation of the cAMP accumulation assay. (f) Representative curves of cAMP accumulation in μOR stably expressing cells following treatment with forskolin (0.5 μM) and increasing concentrations of morphine and buprenorphine. Data in dose–response curves are expressed in % respect to the effect induced by forskolin (100%). (g, h) *E*_max_ (g) and pEC_50_ (h) of the cAMP accumulation assay with morphine and buprenorphine (in means ± S.E.M.; *n* = 7–8, with quadruplicates).

### 3.5 Influence of Morphine and Buprenorphine Binding on the Conformation of the PIF Motif of μOR

The mechanism of agonist-induced receptor activation and G protein binding is accurately characterized.^63^ It includes a polar network in the receptor core (NPxxY and DRY motifs and Y^5.58^) that transmits the signal from the orthosteric ligand binding site to the G protein binding site via the highly conserved PIF motif.^21,23,24^ Basically, the interaction of agonists with TM 5 triggers an inward movement of TM 5 at P^5.50^, a rotation of TM 3 at I^3.40^, and an outward movement of TM 6 at F^6.44^.^64,65^ Thus, we explored the conformation of the PIF motif in the μOR-Gi complex upon binding of morphine, buprenorphine, and the A1 intermediate, which contains all functional groups of buprenorphine except the cyclopropyl group, using MD simulations. We first observed that the buprenorphine binding site is positioned slightly closer to the extracellular side than those of morphine or A1, as indicated by the larger z-coordinate of the N1 atom (Figure 7b). The bulky cyclopropyl group of buprenorphine restricts deep binding, favoring a higher position within the receptor pocket.^66^ Interestingly, A1 lacks the cyclopropyl group yet also binds higher than morphine. We suggest that, in addition to the cyclopropyl group limiting deep binding, buprenorphine extends toward the extracellular side to form favorable hydrophobic interactions between the t-butyl group and L221^45.52^ in ECL 2 (see above).

**Figure 7:**
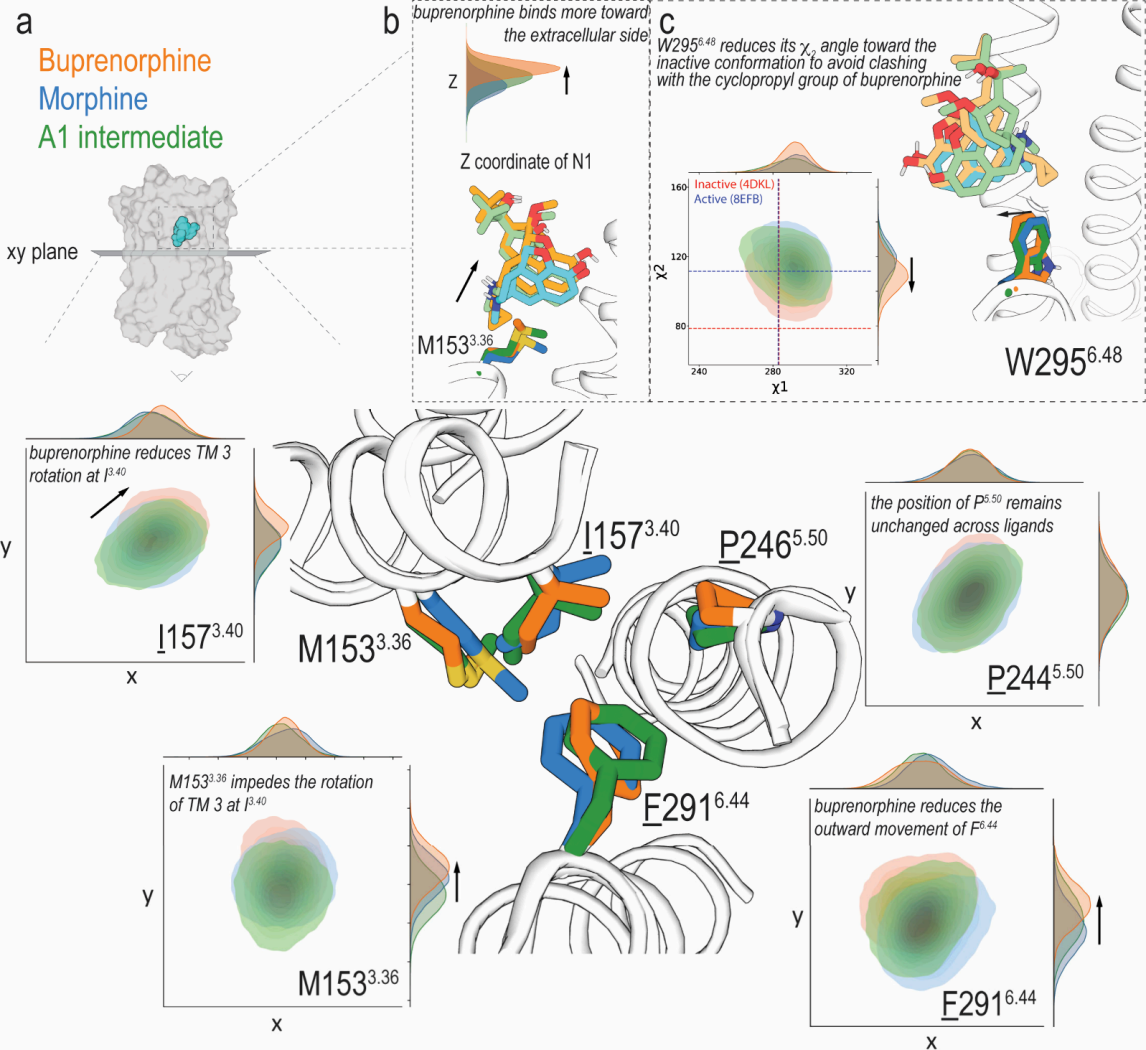
Influence of ligand binding on the conformation of the PIF motif of μOR. (a) Representative conformations of the side chains belonging to the PIF motif (P246^5.50^, I157^3.40^, F291^6.44^) and the toggle switch (M153^3.36^, W295^6.48^) obtained in MD simulations of the μOR-Gi complex in the presence of buprenorphine (orange), morphine (blue), and the A1 intermediate (green). Evolution of the C_α_ atoms of P246^5.50^, I157^3.40^, F291^6.44^, and M153^3.36^ during three replicas of unbiased 1-μs MD simulations and their corresponding distributions. The *xy* plane is defined by the Orientations of Proteins in Membranes (OPM).^34^ Distributions of the *x* and *y* values are shown on the top *x*-axis and right *y*-axis, respectively. Note that the orientation of the molecular models shown in the center was selected for clarity and does not correspond to that of the contour plots. (b) The distributions of the *z*-coordinate of the N1 atom of buprenorphine, morphine, and A1 are shown, with the *z*-axis defined as perpendicular to the *xy* plane. (c) The contour plot of the χ_1_ and χ_2_ dihedral angles of W295^6.48^ is presented, along with the corresponding χ_1_ and χ_2_ values from the inactive 4DKL and active 8EFB structures.

The evolution of the Cα atoms of the amino acids P246^5.50^, I157^3.40^, and F291^6.44^ of μOR during three replicates of unbiased 1 μs MD simulations are shown in Figure 7a. Notably, the conformation of P246^5.50^ in the simulations with morphine, buprenorphine, or A1 is similar. This is reasonable since there are no structural differences in the regions of the ligands that interact with TM 5. In contrast, there are significant differences in the conformation of I157^3.40^. Buprenorphine, unlike morphine or A1, blocks the clockwise rotation of TM 3, as viewed from the intracellular side, due to the insertion of its cyclopropyl moiety. This is reflected in the position of M153^3.36^, whose side chain has also not rotated due to steric hindrance from the cyclopropyl group. Notably, M153^3.36^ and W295^6.48^, located opposite each other in TMs 3 and 6, form the toggle switch that is important in the process of agonist-induced receptor activation.^67^ Figure 7c shows the conformations (χ_1_ and χ_2_ dihedral angles) of W295^6.48^, during the simulations. Clearly, the cyclopropyl moiety modifies the χ_2_ angle of W295^6.48^ toward the inactive conformation. Finally, the outward movement of F291^6.44^ is more pronounced in morphine and A1 than in buprenorphine (Figure 7a). These results suggest that the cyclopropyl moiety of buprenorphine contributes to its partial agonist activity. However, morphine and buprenorphine exert only minor differences in their effects on the PIF motif and toggle-switch residues. NMR spectroscopy has shown that agonist binding, in the absence of G protein engagement, induces only partial or preactive conformations, whereas G protein binding is the primary driver of receptor activation, causing the most significant structural changes.^68^ Accordingly, given that Gi-bound active states were used in the simulations, only subtle changes in μOR conformation were expected upon binding the full agonist morphine and the partial agonist buprenorphine.

We also extracted the structure of 54 ligands classified by ChEMBL^69^ as approved drugs and clinical candidates for μOR.^70^ Among these, 25 compounds possessed the morphinan skeleton; 11 acted as full agonists, whereas 14 functioned as partial agonists or antagonists (Figure 8). Notably, all 11 full agonists exclusively contain a methyl group bound to N17. In contrast, the 14 partial agonists or antagonists contain bulkier substituents such as an allyl group (3 compounds), a cyclopropylmethyl group (9 compounds), or a cyclobutylmethyl group (2 compounds) (Figure 8). Thus, structure–activity relationships confirm that the insertion of bulky moiety into a cavity extending toward the intracellular side is an important structure feature for μOR inactivation.

**Figure 8:**
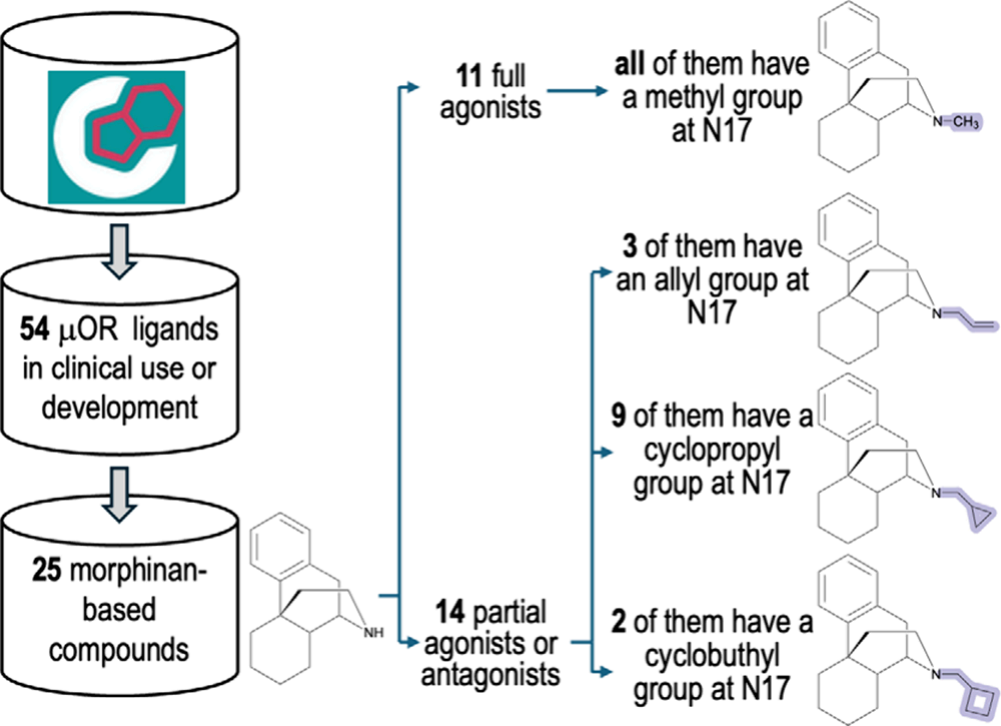
Structure–activity relationships (SAR) of approved drugs and clinical candidates targeting the μOR (ChEMBL233) and opioid receptors (ChEMBL2095181) in *Homo sapiens*, as reported in ChEMBL (accessed October 28, 2025).

## 4 Conclusions

Activity cliffs refer to small structural changes between similar compounds that bind to the same target protein but exhibit a significant difference in biological activity, such as affinity, efficacy, or selectivity.^71,72^ In this way, buprenorphine introduces subtle structural modifications to the morphinan scaffold in comparison with morphine, altering its pharmacological properties. As recently reviewed,^73,74^ buprenorphine acts as a partial agonist at the μOR and as an antagonist at the κOR with similar binding affinities, while acting as a ∼30-fold weaker antagonist at the δOR. Buprenorphine is also a μOR-biased agonist that fails to recruit β-arrestin.^75^ Moreover, morphine and buprenorphine are chiral opioid ligands, with the (−)-enantiomers being pharmacologically active, whereas the (+)-enantiomers are inactive.^76^ Here, we have examined in detail the structural features of (−)-buprenorphine that contribute to its enhanced binding affinity and partial agonism compared to (−)-morphine.

Moreover, extensive evidence from both *in vitro* and *in vivo* studies indicates that the μOR can form heteromeric complexes with a wide range of opioid and nonopioid receptors.^77,78^ For example, the μOR has been reported to heteromerize with orphan GPCRs,^79,80^ as well as with members of both class A^28^ and class B^40^ families. Notably, the galanin Gal_1_ receptor allosterically modulates the efficacy of (*S*)-methadone, but not (*R*)-methadone, thereby reducing its abuse liability.^81^ On this basis, DFNZ, a μOR superagonist analgesic with minimal adverse effects, was recently characterized.^82^ In fact, opioid receptor dimerization has regained relevance, as recent studies using single fluorescent-molecule tracking with TIRF microscopy have demonstrated both dynamic homomerization and heteromerization among the μ, δ, and κ opioid receptors.^53,83^ Each receptor type underwent transient homodimerization or heterodimerization with a specific lifetime of 0.1–0.3 s, depending on the protomer pair, repeatedly dissociating and reassociating with another protomer every few seconds or less. The results of these dynamics studies indicate that opioid receptor monomers predominate at densities around 1 protomers/μm^2^, whereas homomeric and heteromeric assemblies become more prevalent at more probable physiological densities above 10 protomers/μm^2^.^53,83,84^

The competitive binding assay revealed that morphine and buprenorphine display nonclassical μOR binding profiles, indicating the presence of two distinct binding sites on the receptor with differing affinities. As mentioned before, these two binding sites more often indicate different affinities for the two protomers of a GPCR homodimer.^52^ Morphine exhibits negative cooperativity, a classical and commonly observed behavior in GPCR homodimers, whereby binding of a ligand molecule to one protomer reduces the affinity of the same ligand for the second protomer. In contrast, buprenorphine shows positive cooperativity, a much less frequent and nonclassical phenomenon, enhancing the affinity of the same ligand for the second protomer.^85^ These opposite cooperative effects stem from two key features of buprenorphine: the C7 *t*-butyl group induces an inward shift of TM 5 by interacting with L221^45.52^ in ECL 2, while the C6–C14 ether bridge causes steric repulsion with I298^6.51^ and V302^6.55^, inducing an outward displacement of TM 6. Using alchemical free energy methods, we found that substitution of the *t*-butyl group by a hydrogen atom resulted in a free energy penalty of 1.63 kcal/mol (a favorable steric effect for the original *t*-butyl group). Conversely, substitution of the ether bridge favored binding by −1.52 kcal/mol (indicating a steric clash from the original ether bridge). Movements of TMs 5 and 6 in protomers A and B of the μOR are tightly coupled and mutually dependent. Consequently, the initial binding of buprenorphine to protomer A triggers a conformational shift that is transmitted to protomer B via TMs 5 and 6. These movements act as a positive allosteric mechanism, reshaping the protomer B binding cavity to create the optimal fit for buprenorphine (positive cooperativity).

The free energy calculations shows that the 2-hydroxy-3,3-dimethylbutan-2-yl moiety at the C7 position of buprenorphine contributes most to the enhanced binding affinity (2.77 kcal/mol). Moreover, the cyclopropyl moiety of buprenorphine fits into an intracellular cavity (formed by G327^7.42^), stabilizing its interaction with μOR by approximately 1.34 kcal/mol. It has been proposed that these groups are responsible for potency/selectivity (address) and efficacy (message), respectively.^21,86^ To assess the impact of the cyclopropyl group on the efficacy of μOR activation, we examined the conformations of the PIF-motif and toggle-switch side chains using MD simulations of the μOR-Gi complex bound to three ligands: morphine (a full agonist lacking the cyclopropyl moiety), buprenorphine (a partial agonist containing the cyclopropyl moiety), and the A1 intermediate (a negative control structurally similar to buprenorphine but without the cyclopropyl group). Morphine and A1 display comparable conformational changes in both the PIF motif and the toggle switch. In contrast, buprenorphine restricts the rotation of TM 3 and alters the conformation of W295^6.48^, thereby stabilizing a more inactive receptor state.^87^

Understanding and dissecting the specific functional groups of buprenorphine that underlie its beneficial pharmacological properties is essential, as identifying these molecular determinants provides a rational framework for the design of next-generation ligands with improved safety profiles. Furthermore, receptor heteromerization may contribute to buprenorphine’s distinct pharmacological properties and is currently under investigation.

## Supplementary Material



## Data Availability

Input coordinates, topology files, ligand parameters, input files, and the trajectories collected during 3 replicas of MD simulations (in a PyMol session) of morphine, buprenorphine, and the A1 intermediate bound to the μOR-Gi complex and the μOR-μOR homodimer are available at https://zenodo.org/uploads/18863768. Files for minimization, equilibration, and production steps of the TI simulations in the protein–ligand complex and in solution are also provided. PACKMOL-Memgen and cpptraj, distributed with AmberTools, is free of charge; MDAnalysis and GROMACS are open source; AMBER and VMD are available to noncommercial users under a distribution-specific license; and PyMOL is a commercial software with a paid license.

## Notes

Work distributions during the nonequilibrium alchemical transformations (PDF)

## Abbreviations

EM1: endomorphin-I
FDA: Food and Drug Administration
GPCR: G protein-coupled receptor
MD: molecular dynamics
μOR: μ-opioid receptor
NanoBiT; NanoLuc Binary Technology; OPM: Orientations of Proteins in Membranes
OUD: Opioid Use Disorder
PROSPECT: Procedure-Specific Postoperative Pain Management
RMSD: root-mean-square deviation
SAR: Structure–Activity Relationships
TI: thermodynamic integration
TIRF: total internal reflection fluorescence microscopy
TM: transmembrane
WHO: World Health Organization
