# Supplementary material for: Emerging Insights into the Distinct Pharmacological Mechanisms of Buprenorphine
Source: J Chem Inf Model. 2026 May 20;66(11):6733–45. doi: 10.1021/acs.jcim.6c00672 (PMC13250898; doi:10.1021/acs.jcim.6c00672)
Supplement: Supplementary file 1 [file ci6c00672_si_001.pdf]

Emerging Insights into the Distinct Pharmacological Mechanisms of Buprenorphine

Aràntzazu Alonso-Carrasco[a,#], Aleix Quintana-Garcia[b,#], Marc Ciruela-Jardí[a,b,#], Verònica Casadó-Anguera[a,#], Garett A. Enten[c], Natàlia Llopart[a], Berta Carrasco-Martinez[b], Ning-Sheng Cai[c], Estefanía Moreno[a], Sergi Ferré[c], Vicent Casadó[a,*], Leonardo Pardo[b,*]

[a]Laboratory of Molecular Neuropharmacology, Department of Biochemistry and Molecular Biomedicine, Faculty of Biology and Institute of Biomedicine, University of Barcelona, Barcelona, Spain

[b]Laboratory of Computational Medicine, Biostatistics Unit, Faculty of Medicine, Autonomous University of Barcelona, Bellaterra, Spain

[c]Integrative Neurobiology Section, National Institute on Drug Abuse, Intramural Research Program, National Institutes of Health, Baltimore, MD, USA

#contributed equally

KEYWORDS

Morphine, buprenorphine, radioligand binding assays, positive cooperativity, alchemical free energy methods, MD simulations, cAMP, NanoBiT

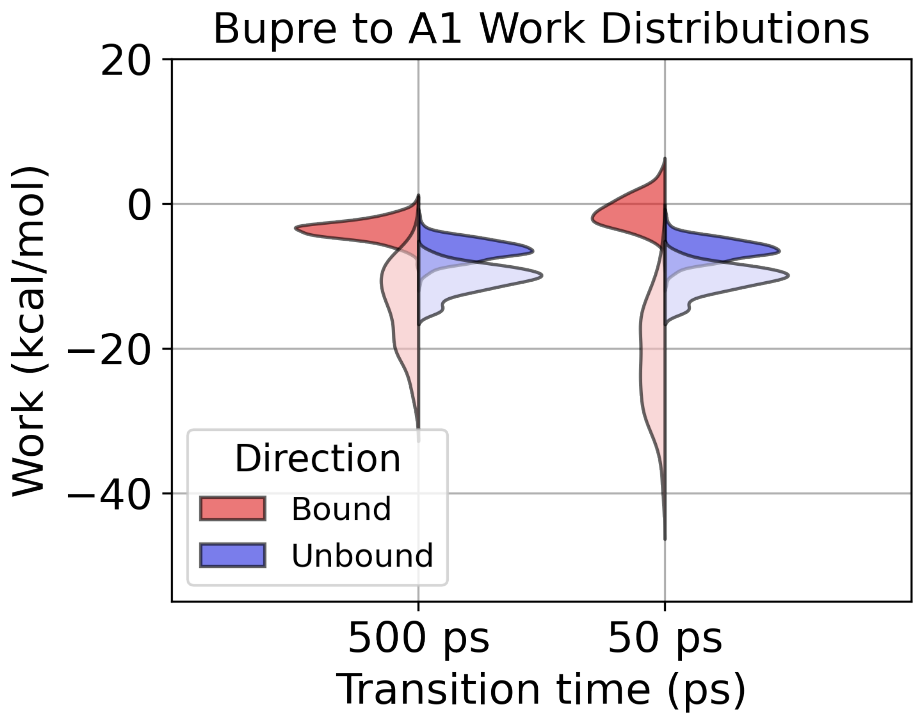

Figure S1. Comparison between buprenorphine to intermediate A1 work distributions during the non-equilibrium alchemical transformations using switching times of 50 ps and 500 ps. Calculations performed using μOR are presented in red, whereas calculations performed in aqueous solution are shown in blue. The forward (A→B) and backward (B→A) transformations are shown in darker and lighter colors, respectively. Forward and backward work distributions in μOR using a 50 ps switching time show almost no overlap, leading to significant numerical inaccuracies in the estimation of ΔG. When the switching time is increased to 500 ps, the two distributions come closer together, resulting in a more reliable estimation of the intersection point of the forward and reverse work distributions, which is used to calculate ΔG. Using a 500 ps non-equilibrium switching time yields a $\Delta G_{A\rightarrow B}$ of −6.89 ± 0.99 kcal/mol, compared to −7.39 ± 1.59 kcal/mol as reported by the built-in pmx *analyze* tool.

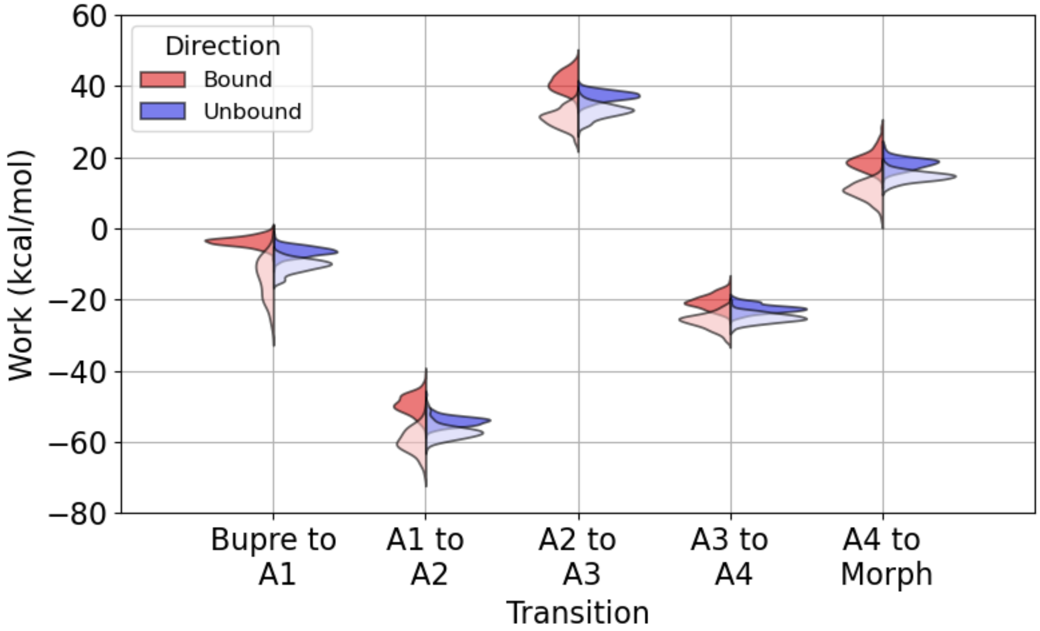

Figure S2. Alchemical free energy calculations. Calculations performed using μOR are presented in red, whereas calculations performed in aqueous solution are shown in blue. The forward (A→B) and backward (B→A) transformations are shown in darker and lighter colors, respectively. The free energy difference for a given transformation ($\Delta G_{A \rightarrow B}$) is estimated as the intersection of the forward and backward work distributions, as stated by the Crooks Fluctuation Theorem. Forward and backward transitions follow Gaussian distribution and overlap appropriately for all cases. Overlap is greater in the unbound than the bound state, as one would expect.
